# Supplementary material for: Inference of Gene Regulatory Network Uncovers the Linkage between Circadian Clock and Crassulacean Acid Metabolism in Kalanchoë fedtschenkoi
Source: Cells. 2021 Aug 27;10(9):2217. doi: 10.3390/cells10092217 (PMC8471846; doi:10.3390/cells10092217)
Supplement: Supplementary file 1 [file cells-10-02217-s001.zip › Figure_S1.pdf]

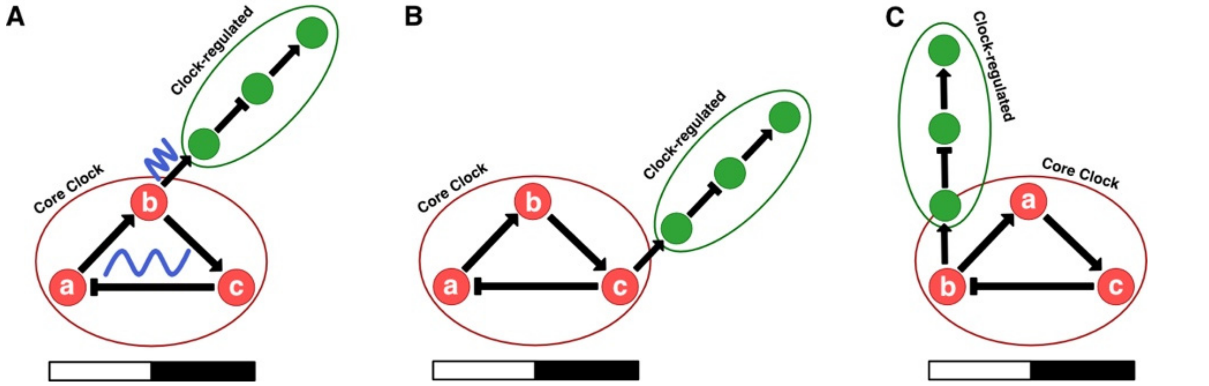

Figure S1. Models for circadian clock-related alterations of physiology at the molecular level. **(a)** The core clock generates waves of transcription that propagate into connected (clock-regulated) gene regulatory networks (GRNs) **(b)** GRNs can be rewired to different core clock genes **(c)** Core clock genes can be rewired within the core clock network, carrying the GRNs they are connected to with them. Red circles are core clock genes. Green circles are clock-regulated genes. White and black bars indicate daytime (12-hour) and nighttime (12-hour), respectively.
