# Supplementary material for: Inference of Gene Regulatory Network Uncovers the Linkage between Circadian Clock and Crassulacean Acid Metabolism in Kalanchoë fedtschenkoi
Source: Cells. 2021 Aug 27;10(9):2217. doi: 10.3390/cells10092217 (PMC8471846; doi:10.3390/cells10092217)
Supplement: Supplementary file 1 [file cells-10-02217-s001.zip › Figure_S2.pdf]

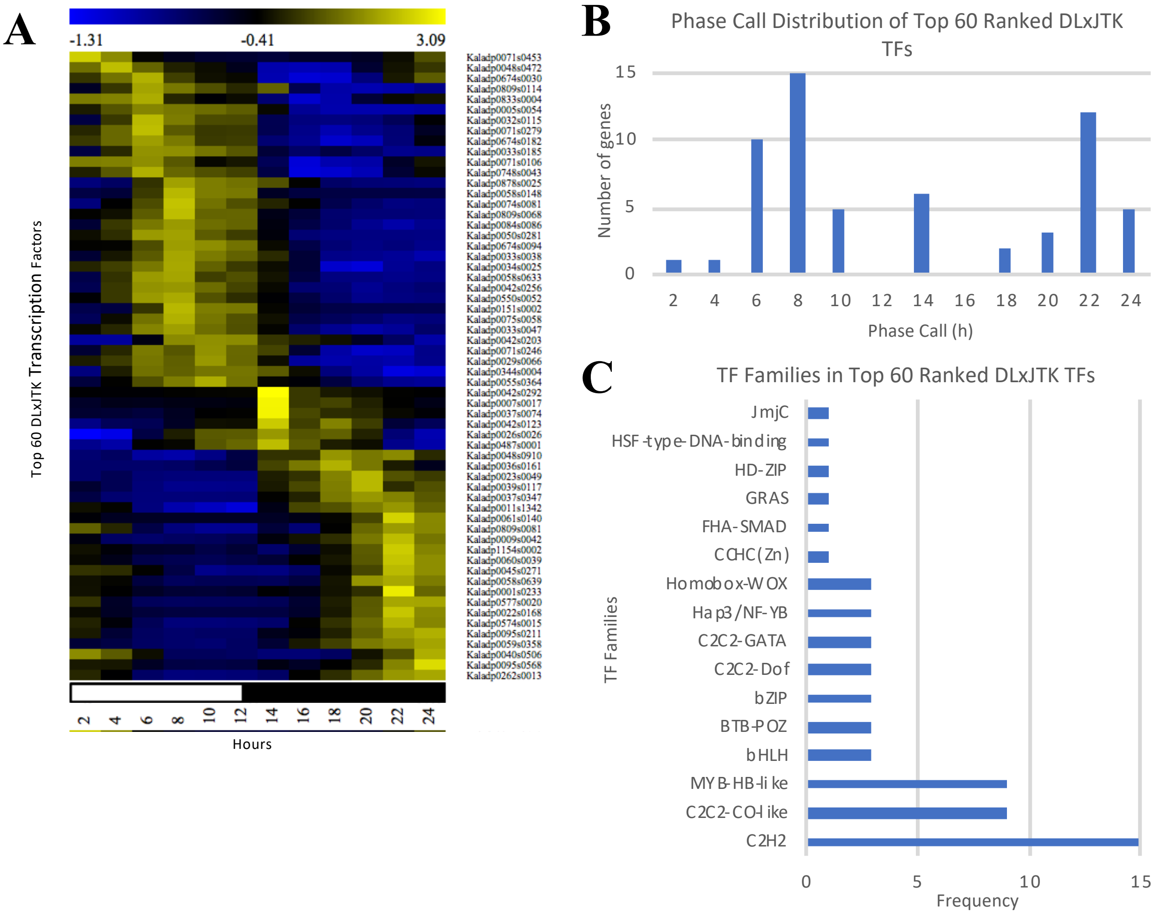

Figure S2. Assessment of transcription factors identified by DLxJTK.
(a) Heatmap displaying z-score standardized expression profiles of the top 60 transcription factors (TFs) pulled from DLxJTK ranked ordered gene list. White and black bars indicate daytime (12-hour) and nighttime (12-hour), respectively. **(b)** Phase call distributions of the top 60 TFs identified with DLxJTK. **(c)** Frequency of TF families in the top 60 TFs identified with DLxJTK.
