# Supplementary material for: Inference of Gene Regulatory Network Uncovers the Linkage between Circadian Clock and Crassulacean Acid Metabolism in Kalanchoë fedtschenkoi
Source: Cells. 2021 Aug 27;10(9):2217. doi: 10.3390/cells10092217 (PMC8471846; doi:10.3390/cells10092217)
Supplement: Supplementary file 1 [file cells-10-02217-s001.zip › Figure_S3.pdf]

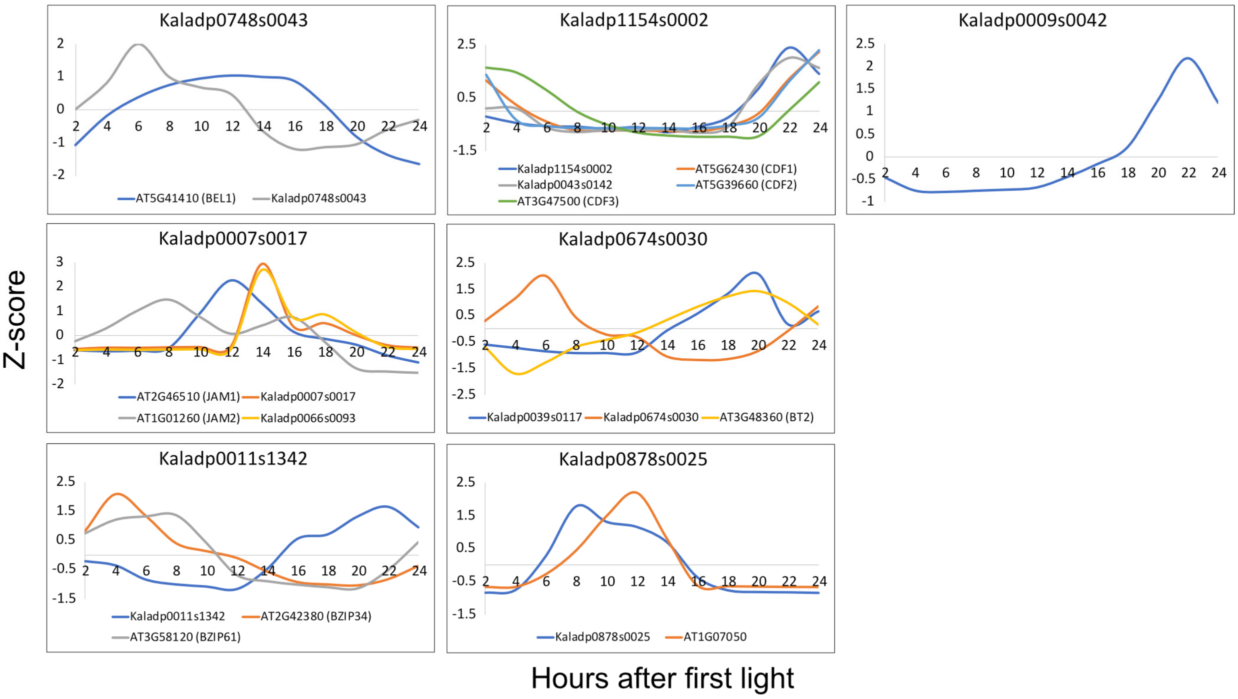


**Figure S3.** Kalanchoë fedtschenkoi candidate clock transcription factors and their Arabidopsis thaliana orthologs. Z-score standardized expression profiles of the K. fedtschenkoi candidate clock transcription factors, as well as their respective paralogs and A.s thaliana orthologs if present.
